# Electrochemical Behavior of SiC-Coated AA2014 Alloy through Plasma Electrolytic Oxidation

**DOI:** 10.3390/ma15103724

**Published:** 2022-05-23

**Authors:** Talal A. Aljohani, Majed O. Alawad, Sally Elkatatny, Abdulrahman I. Alateyah, Meteb T. Bin Rubayan, Mohammed A. Alhajji, Muntathir I. AlBeladi, Fuad Khoshnaw, Waleed H. El-Garaihy

**Affiliations:** 1 Materials Science Research Institute, King Abdulaziz City for Science and Technology (KACST), Riyadh 12354, Saudi Arabia; moalawad@kacst.edu.sa (M.O.A.); mbinrubayan@kacst.edu.sa (M.T.B.R.); malhajji@kacst.edu.sa (M.A.A.); mialbeladi@kacst.edu.sa (M.I.A.); 2 Mechanical Engineering Department, Faculty of Engineering, Suez Canal University, Ismailia 41522, Egypt; sally_mahmoud@eng.suez.edu.eg (S.E.); or w.nasr@qu.edu.sa (W.H.E.-G.); 3 Department of Mechanical Engineering, College of Engineering, Qassim University, Unaizah 56452, Saudi Arabia; a.alateyah@qu.edu.sa; 4 School of Engineering and Sustainable Development, De Montfort University, Leicester LE1 9BH, UK; fuad.hassankhoshnaw@dmu.ac.uk

**Keywords:** plasma electrolytic oxidation, AA2014 aluminum alloy, potentiodynamic polarization, electrochemical impedance spectroscopy (EIS)

## Abstract

In this study, the corrosion performance of AA2014 aluminum alloy was enhanced by coating the alloy with a layer containing silica (SiC) that was formed by the plasma electrolytic oxidation (PEO) process. The PEO process was performed with different electrical parameters (frequency, current mode, and duty ratio) and both with and without SiC to investigate the microstructural and electrochemical differences in the coated samples produced from the process. The microstructure and composition of the PEO coatings were studied using X-ray diffraction (XRD) and scanning electron microscopy (SEM) with energy dispersive spectroscopy (EDS). A potentiodynamic polarization test and electrochemical impedance spectroscopy (EIS) were used to investigate the electrochemical behavior of the AA2014-PEO-coated samples. The potentiodynamic polarization showed that the SiC-PEO-coated samples had a significantly decreased corrosion rate (99.8%) compared with the uncoated AA2014 Al alloy. Our results showed that the coats containing SiC possessed a much higher corrosion resistance than both the uncoated AA2014 Al alloy (8,344,673%) and the SiC-free coatings, which possess low corrosion resistance, because of their higher chemical stability and more compact microstructure.

## 1. Introduction

Aluminum (Al) and Al-based alloys have desirable characteristics, such as a high strength/weight ratio, good formability, and nonmagnetic properties [1,2,3]. Naturally, this makes Al and Al alloys appealing to a wide variety of industries, especially automotive and aerospace industries. However, Al and its alloys have low hardness, low wear resistance characteristics, and poor corrosion properties, which have severely restricted their potential for use in a wide range of applications [3,4,5,6,7]. Furthermore, the natural oxide film present on the Al surface does not offer sufficient preservation from aggressive anions [8]. Based on these findings, the key to improving Al alloys for industry use in different applications is improving their mechanical properties and corrosion behavior.

Among Al alloys, the industry’s research efforts have been mostly focused on studying Al–Cu (AA2xxx) alloys due to their superior properties for structural applications such as those pertaining to spacecraft [9,10,11,12,13,14,15]. Nevertheless, it was reported that adding Cu decreases the corrosion resistance of the following alloys: Al + 1–2% Cu; Al + 5% Cu; age-hardenable alloys AA7475-T761, AA7075-T651, AA2024-T4, and 2011-T3 [16]. This decrease occurs due to the formation of an intermetallic compound (CuAl_2_) during heat treatment. CuAl_2_ encourages corrosion, and acts as a cathode inclusion in this process. Thus, it has become vital to investigate and develop corrosion protection methods for Al–Cu alloys. Numerous studies have been conducted to enhance the several properties mentioned above employing surface treatments. Li et al. [17] observed that laser surface melting of AA 2024-T351 alloy using a CO_2_ laser improved the microstructure of the surface layer without impacting its pitting potential. Finally, laser surface melting of AA 2014-T6 alloys raised the pitting potential in 3 wt.% NaCl solution and altered the pitting potential to 170 mV [18].

Plasma electrolytic oxidation (PEO) is a surface treatment technique famous for being environmentally friendly. When applied to lightweight alloys, such as Al, Mg, and Ti alloys, it improves their wear and corrosion resistance by creating a moderately hard, dense, and thick coating [19,20,21,22,23]. However, due to the advances in industrial technologies, the performance requirements for Al alloys have become more demanding. As a result, improving the PEO coatings to meet industry standards has become a challenge to scientists. PEO coatings’ properties are primarily caused by their structural characteristics [24]. The properties and performance of PEO coatings depend on the substrate alloying elements of the electrolytic chemistry and the value and mode of the applied current density, as these parameters directly affect the compound, porosity, and thickness of the coating. PEO coatings were also used to improve the poor localized and pitting corrosion resistances of AA 6061 alloys when Al_2_O_3_ nanoparticles were added to the electrolyte [25]. 

The properties of Al–Cu alloys, such as corrosion resistance, thermal conductivity, and wear, can be improved by adding appropriate nanopowders to the PEO electrolyte that is used [26,27,28,29]. However, little effort has been exerted in the past few years to enhance Al–Cu alloys’ coatings’ properties. Moreover, few researchers have attempted to investigate the electrochemical, tribological, and mechanical effects of adding nanoparticles to PEO coatings processed using either different electrolytes or different current densities. Thus, in this study, we aimed to enhance the corrosion behavior of AA2014 alloy by adding SiC particles through PEO. In addition, the effect of electrical parameters on the PEO process and the coated layers that form on AA2014 alloys is presented; we investigated the corrosion behavior and microstructural characteristics through different PEO electrical parameters.

## 2. Materials and Methods

In this study, an AA2014 alloy with a chemical composition of 4.4 wt% Cu, 0.8 wt% Si, 0.5 wt% Mg, and 93.5 wt% Al was used. Initially, rectangular samples with dimensions 110 × 45 × 10 mm were cut and polished with 600, 800, 1200, and then 4000 grit silicon carbide papers. Then, the samples were cleaned with acetone in an ultrasonic bath and rinsed with deionized water. Finally, samples were directly subjected to the PEO process without a pre-heat treatment.

The PEO process was performed in an alkaline solution that contained 3 g/L of potassium hydroxide (KOH) and 2 g/L of sodium silicate (Na_2_SiO_3_). Moreover, 1 g/L of SiC was added to the alkaline solution of some of the samples. The anode was connected to the sample; the cathode was connected to a polyvinyl chloride container lined with a 1 m^2^ stainless steel sheet. A cooling system was connected to the cell to retain the temperature at about 25 °C. The solution was continuously mixed using air agitation.

The experimental procedures were conducted on unipolar (UP) and bipolar (BP) samples with different combinations of duty cycles and frequencies to investigate how the PEO process’s polarity affected the coating’s thickness and structure. The duration of each procedure was 300 s. The designation of the samples consisted of two alphabet letters, denoting the polarity and two numbers denoting the duty cycle, as illustrated in Table 1 The studied duty cycles were calculated based on Equation (1):

(1) $$duty\;cycle=\frac{t_{on}}{t_{on}+t_{off}}$$

where *t_on_* is the on-time, *t_off_* is the off-time, and (*t_on_*+ *t_off_*) is the total time of the procedure.

The electrochemical corrosion tests were performed using a flat corrosion cell with three electrodes and a Bio-Logic SP-200 potentiostat (Lambda System Kreft Barszczewski Sp.J., Warszawa, Poland). Saturated calomel reference electrodes and platinum counter electrodes were used in this setup. A potential scan rate of 0.166 mVs^−1^ was applied in the polarization techniques used to ensure steady-state conditions. Linear polarization scans were conducted with a potential variation (E_range_) of ±25 mV. The EIS tests were employed at open-circuit potential (E_corr_) using a sinusoidal voltage range of ±10 mV and with scan frequencies of 10 mHz to 100 kHz. The electrochemical measurements were conducted in an aerated 0.5 M sodium chloride (NaCl) solution at 298 K.

A JSM-IT300 InTouchScope™ scanning electron microscope (SEM) combined with an X-MaxN Oxford energy-dispersive X-ray spectroscopy (EDS) analyzer was used to explore the surface morphology of the corroded specimens. To investigate the structure’s crystallinity, X-ray diffraction (XRD) measurements were carried out using an XRD type JEOL JDX-8030 X-ray diffractometer, which uses Cu-Kα radiation, operates at 40 kV and 30 mA, and has a scan rate of 2°/min.

## 3. Results and Discussion

### 3.1. Phase Composition and Microstructure Morphology of the Samples

**Figure 1:**
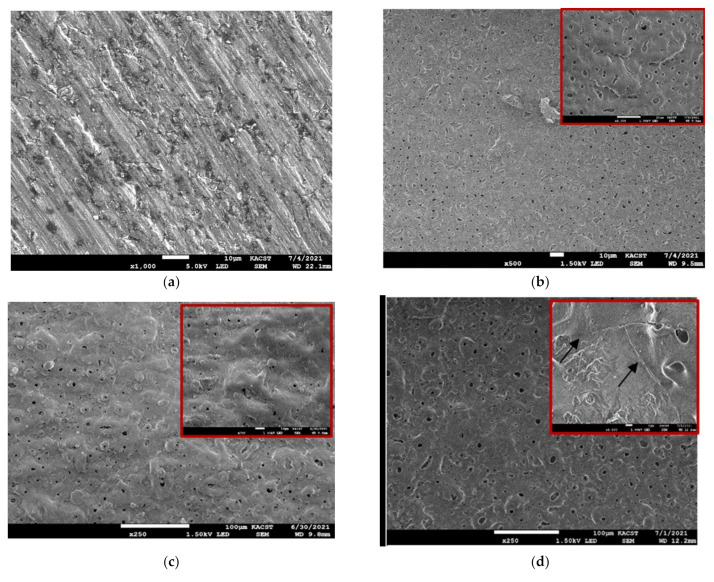

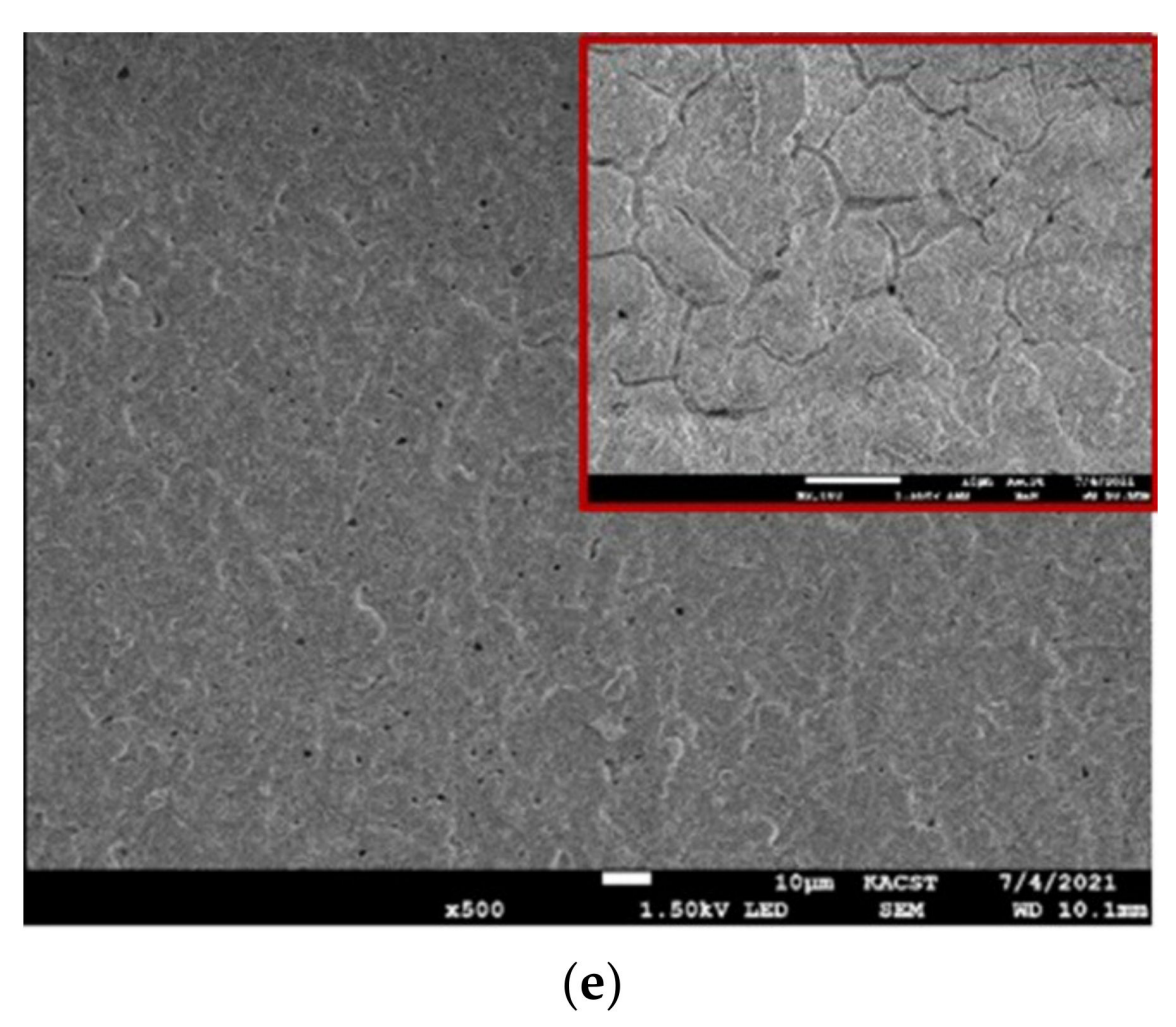
SEM micrographs of AA2014 Al alloy samples subjected to PEO coating under different conditions: (**a**) BM, (**b**) BP50, (**c**) SiC/UP30, (**d**) SiC/BP50_1000, and (**e**) SiC/BP50_2500.

A compilation of the various morphologies of the PEO-coated surfaces of the samples is shown in Figure 1. All the PEO films had micropores on their surface, and differed in size and density from sample to sample. The micropores is mainly formed owing to the gas bubbles generated from the discharge channels and the molten oxide. A close inspection of the surface morphologies of the various AA2014 PEO coatings (Figure 1) yielded the observation that changing the duty cycle, current mode, and frequency parameters resulted in pores with irregular size, shape, and density.

The variation in surface morphology (pore density and size) between samples revealed that the addition of SiC to the base alkaline solution during PEO had a significant impact on the pore morphology, as shown in Figure 1b,d. This stemmed from the surface morphology’s dependency on the electrolyte’s electrical conductivity [25,30]. Adding SiC and selecting the unipolar current mode with a duty cycle of 30% to SiC/UP30 (Figure 1c) led to the formation of moderately large pores with a lower pore density. Nevertheless, coating with the bipolar current mode and increasing the duty cycle to 50% in SiC/BP50_1000 reduced the density and size of the pores compared with those produced with the unipolar mode, as shown in Figure 1d. As previously described, the pores’ characteristics depended on the final voltage. The higher the end voltage applied, the larger the spark on the SiC coated surface. In addition, the initial cracks on the PEO coating surface penetrated the surface due to the high thermal stresses [31]. The final voltage is therefore considered a significant parameter in the PEO-coated surface’s morphology. Additionally, the surface of the AA2014 SiC/BP50_2500 sample contained far more uniformly distributed pores with both smaller sizes and a much higher pore density compared with the coatings processed at a lower frequency (the SiC/BP50_1000 sample), as shown in Figure 1d. The large number of micropores that formed on the surface could be attributed to electrolyte vapors and/or oxygen trapping and growth.

**Figure 2:**
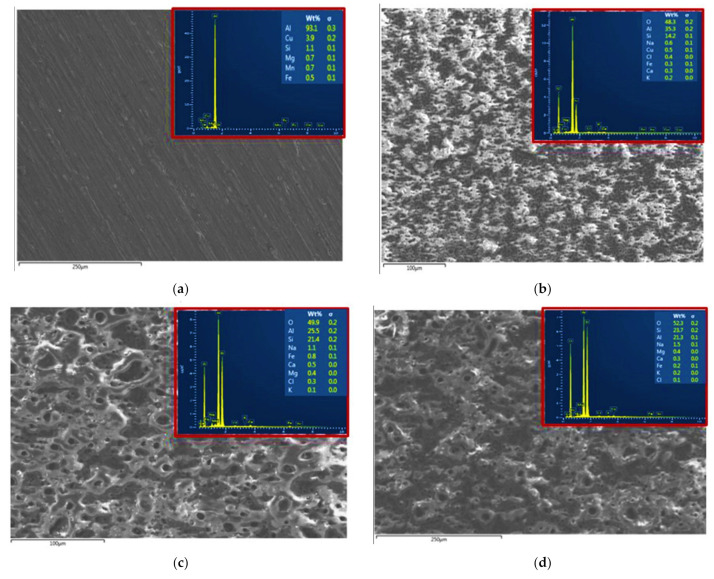

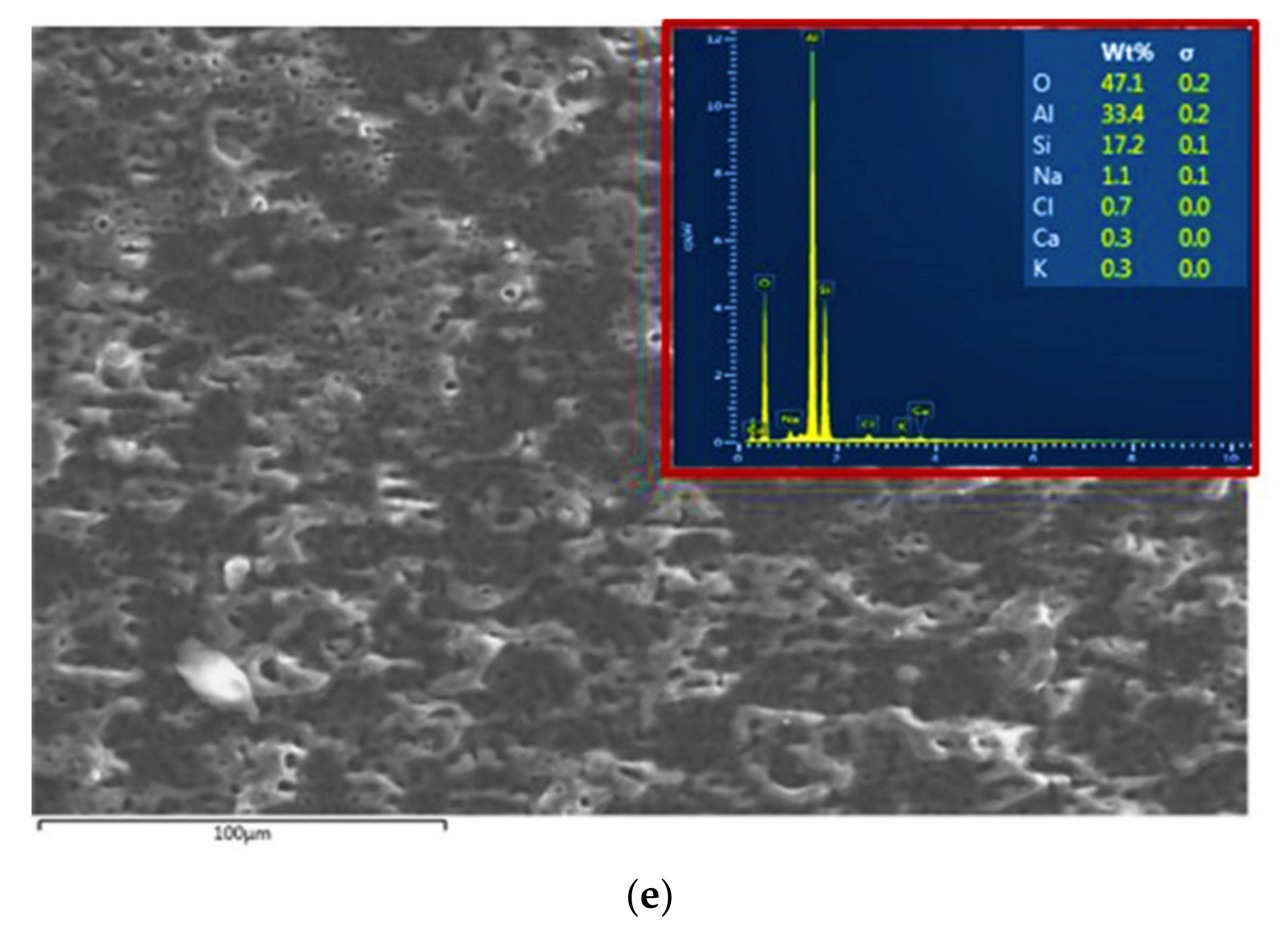
EDS analysis of the AA2014 Al alloy samples after PEO coating under different conditions: (**a**) BM, (**b**) BP50, (**c**) SiC/UP30, (**d**) SiC/BP50_1000, and (**e**) SiC/BP50_2500.

**Figure 3:**
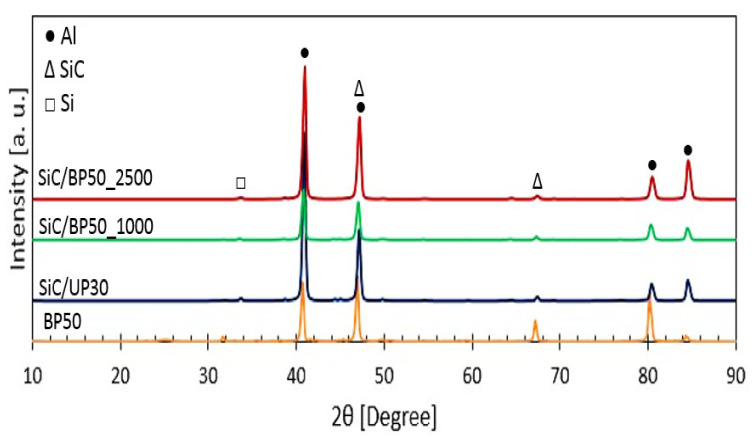
XRD patterns of the AA2014 Al alloy samples’ substrate after coating under different conditions.

The EDS elemental mapping analyses of the AA2014 Al alloy samples are summarized in Table 2 and presented in Figure 2. The previous data revealed that the chemical composition of the PEO coatings was predominantly dependent on the process parameter. The EDS analysis showed that Si and O were the major constituents of the coated samples. As shown in Table 2, higher current densities caused an increase in Si and O percentages. The XRD patterns of the AA2014 PEO-coated samples, processed under different conditions, are presented in Figure 3. The patterns show that the PEO coatings that formed on the studied AA2014 substrate predominantly consisted of SiC with an absence of Al–Cu, in agreement with the EDS results.

**Figure 4:**
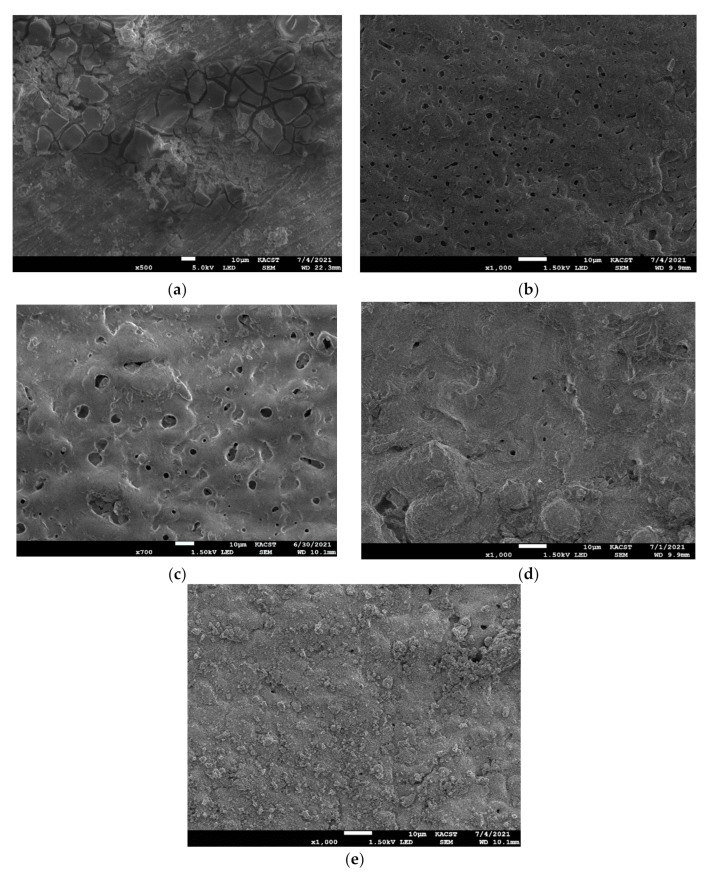
SEM micrographs after conducting the corrosion test of the AA2014 samples subjected to PEO coating under various conditions: (**a**) BM, (**b**) BP50, (**c**) SiC/UP30, (**d**) SiC/BP50_1000, and (**e**) SiC/BP50_2500.

**Figure 5:**
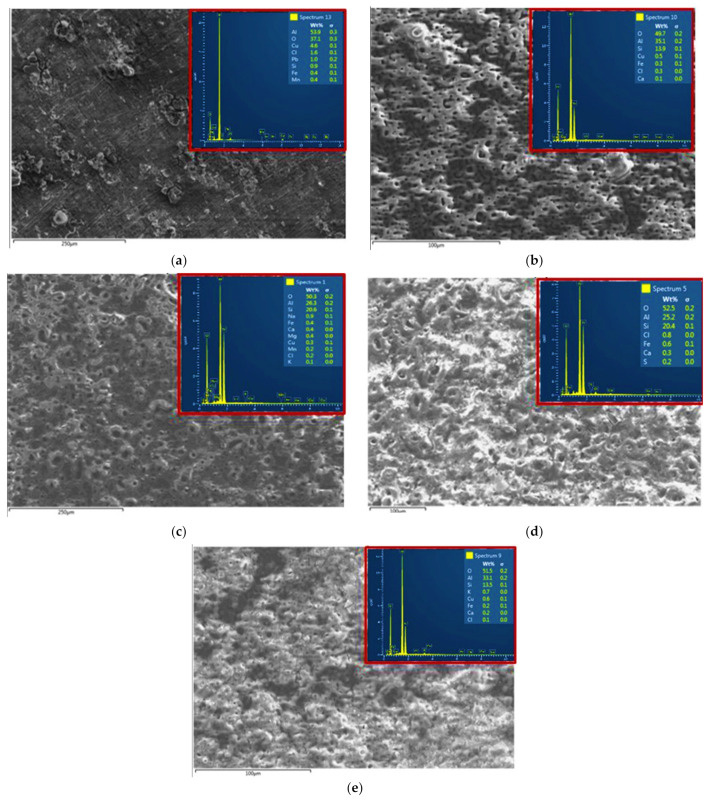
SEM images and EDS analysis of PEO-coated AA2014 samples after the corrosion test: (**a**) BM, (**b**) BP50, (**c**) SiC/UP30, (**d**) SiC/BP50_1000, and (**e**) SiC/BP50_2500.

SEM micrographs of the AA2014 alloy’s BM and coated samples after conducting the corrosion test are shown in Figure 4. The BM sample had the worst protection against corrosion, as it contained numerous cracks and pits (Figure 4a). The BP50 sample, which had no SiC (Figure 4b), had fewer pores and cracks than the BM sample. However, the addition of SiC during PEO coating definitively limited the number of corrosion pits and cracks on the coated samples compared with the other two samples (Figure 4c–e). This improvement suggests that the three SiC-coated samples had excellent corrosion resistance. The corrosion resistance capabilities of the SiC PEO coatings can be evaluated using the samples’ porosity density, as seen in our previous results. The deeply penetrating pores provide the corrosive solution easy access to the metal substrate, thus accelerating the solution’s penetration of the substrate and consequently degrading the anticorrosion performance of the sample [32]. Of the SiC-coated samples, AA2014/SiC_2500 had the best protection, as it contained the lowest count of pores compared with the coatings produced at lower frequencies (SiC/BP50_1000 and SiC/UP30). The composition of the various AA2014-coated samples after corrosion testing is shown in Figure 5. The BM and BP50 samples were primarily composed of Al and O, whereas the SiC/UP30, SiC/BP50_1000, and SiC/BP50_2500 samples were composed of Al, O, and Si, as shown in Figure 5.

### 3.2. Electrochemical Measurements

**Figure 6:**
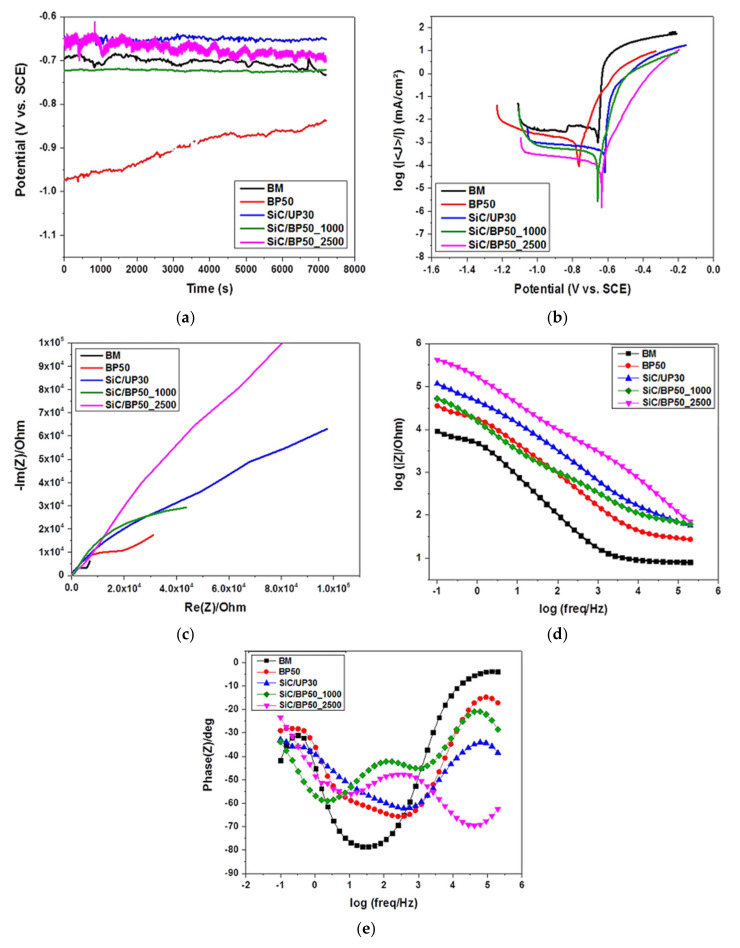
Corrosion measurement curves of PEO-coated AA2014 Al samples: (**a**) open circuit potential, (**b**) potentiodynamic polarization curves, (**c**) Nyquist plots, (**d**) electrochemical impedance diagrams (Bode plot), and (**e**) phase angles.

The open circuit potential (OCP) for the AA2014 samples both before and after coating was plotted against immersion time, and is shown in Figure 6a. All the coated AA2014 samples exhibited shifting in the corrosion potential (E_corr_) toward the positive side, except for the sample coated without SiC (BP50). This shift indicates more passivity in the surface. The AA2014 BM sample did not exhibit any alterations in OCP, while the SiC/BP50_2500 sample showed the largest fluctuations in OCP. However, the BP50 sample had the largest shift in E_corr_ in the negative direction of potential. These fluctuations in potential could be traced back to the porosities present in the coating’s surface.

The potentiodynamic polarization curves for all studied samples are depicted in Figure 6b. The curves presented in Figure 6b show a significant improvement in the corrosion resistance of the AA2014 samples that were coated with SiC. In addition, Figure 6b indicates that the SiC PEO coating process reduced the current density. The SiC/BP50_2500 sample recorded the lowest current density of all the samples. The corrosion current densities (I_corr_) were extrapolated from the Tafel plots; their values are listed in Table 3, alongside both the E_corr_ and the anodic (βa) and cathodic (βc) Tafel constants of the plots. The tabulated data show that the AA2014 BM sample had the maximum I_corr_, which means that the PEO coating caused a decline in the I_corr_ of the substrates regardless of coating conditions. The BP50, SiC/UP30, SiC/BP50_1000, and SiC/BP50_2500 samples experienced reductions in I_corr_ of 72.6%, 88%, 95.7%, and 93.5%, respectively, compared with that of the BM sample. Finally, the SiC/BP50_2500 sample recorded the lowest I_corr_ value, decreasing from 2.276 µAcm^−2^ in the BM sample to 0.098 µAcm^−2^. This drastic decline indicates that the SiC/BP50_2500 coating had the maximum corrosion resistance of all the coatings.

To confirm whether SiC–based coatings are predisposed to pitting corrosion, EIS was performed on all the studied samples’ conditions in a 0.5 M NaCl solution. EIS is moderately helpful for quantifying aggressive ion adsorption at electrode interfaces. The impedance spectra achieved with complex impedance (Nyquist diagram), the electrochemical impedance (Bode amplitude), and the phase angle plots are illustrated in Figure 6c, Figure 6d, and Figure 6e, respectively. The presence of diffusion elements indicates the adsorption of anions at the metal–solution interface [33]. The mechanism of pitting initiation in Al and its alloys involves three steps: First, chloride ions are absorbed and penetrate the oxide surface. Second, soluble hydroxy chloride Al salt is formed. Finally, the oxide dissolves in the regions where the film is thinner [34,35,36].

The Nyquist plots in Figure 6c reveal that the SiC-based PEO coating caused an increase in the diameters of the AA2014/SiC coated samples’ semicircle, which demonstrates the improvements in their corrosion protection. The SiC/BP50_2500 sample exhibited the largest semicircle diameter of all the coated samples. This increase in diameter indicates that the sample had the best corrosion resistance of all the samples, which is in good agreement with the after-corrosion SEM micrographs (Figure 4). Moreover, the Bode plots presented in Figure 6d clearly show that the samples’ corrosion resistance was improved by SiC-based PEO treatment. The SiC/BP50_2500 sample exhibited the largest impedance magnitudes (|Z|) at all the tested frequencies, whereas the BP50 (SiC-less) sample demonstrated smaller impedance magnitudes than the SiC/UP30 sample (Figure 6d). All SiC-coated samples showed comparable impedance magnitudes of nearly twice that of the BM impedance at higher frequencies, as shown in Figure 6d. In addition, Figure 6e shows the phase angles of AA2014 after PEO coating. The SiC/BP50_2500 sample had a phase angle of almost −70° at the highest frequencies, which shows that the coating resulted in better corrosion properties. Finally, the corrosion mechanisms of the studied coatings at the electrode–electrolyte interface were explored using EC’s labV10.37 software and the Randomize+ simplex method.

**Figure 7:**
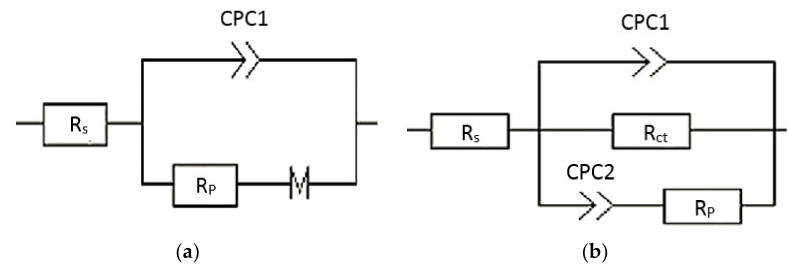
Equivalent circuit models for (**a**) AA20214BM and (**b**) PEO-coated samples.

The EIS data for the AA2014 base metal and the samples PEO-coated with different conditions were fitted using equivalent circuit (EC) models. The EC models of the AA2014-BM and other studied conditions are shown in Figure 7a,b, and are listed in Table 4. The EC for AA20214BM consists of a solution resistor [R_s_] connected in series to a constant phase component [CPC1] and a charge transfer resistance [R_ct_], both of which are connected in parallel [37] (Figure 7a). [R_s_] represents the solution’s corrosion resistance, while CPC represents a double-layer capacitor. The EC model of the AA2014 PEO-coated samples consisted of a solution resistance [R_s_], a polarization resistance [R_p_], an R_ct_, and two constant phase components (CPC1 and CPC2) (Figure 7b). In that EC, the CPC2 and R_ct_ are the inner layer of coatings.

Analysis of the EC of the AA2014 coated samples (Table 4) revealed significantly larger R_ct_ values compared with the R_p_. In addition, Table 4 confirms that the SiC/BP50_2500 sample showed the best improvement in corrosion performance. This improvement was reflected in the substantial amplification of the R_ct_, which increased from 7.902 (BM) to 659,404 Ω·cm^2^; this enhancement in corrosion protection could arguably be linked to the inner coating layer. Finally, applying a bipolar current mode (SiC/BP50_1000) resulted in a denser coating and a higher polarization resistance, but at the cost of a lower corrosion current density than that produced by the unipolar current mode (SiC/UP30).

## 4. Conclusions

In this study, surface modification of various aluminum alloy AA2014 samples was performed through plasma electrolytic oxidation (PEO) to analyze the effect of varying the PEO parameters on the microstructural and corrosion properties of AA2014. The coating process took place in potassium hydroxide and sodium-silicate-based electrolytes; furthermore, some of the samples were coated using PEO with the addition of SiC particles, and others were not. The efficiency and performance of the AA2014 coated surface were affected by process parameters, which was evidenced by their variation from sample to sample as different current modes, frequencies, and duty cycles that were applied to the alloy produced different results. Coating with SiC using the bipolar current mode caused a reduction in the pores’ sizes and density compared with both coating without SiC and coating with SiC using the unipolar mode, thus showing that the SiC-bipolar mode enhances the alloy’s corrosion resistance. We concluded from the results that the PEO coatings processed with SiC-based electrolytes resulted in a higher corrosion resistance than those processed in SiC-free electrolytes due to the anticorrosion behavior of the SiC.

## Figures and Tables

**Table 1:** The applied parameters, frequencies, and currents of AA2014.

Sample Designation	Polarity	Frequency (Hz)	Off/On (ms)	Duty Cycle %	Working Electrode (mA/cm^2^)		Time (s)
BP50	Bipolar	2500	0.2/0.2	50	+0.30	−0.1	440
SiC/UP30	Unipolar	1000	0.7/0.3	30	+0.30	−0.1	440
SiC/BP50_1000	Bipolar	1000	0.5/0.5	50	+0.30	−0.1	440
SiC/BP50_2500	Bipolar	2500	0.2/0.2	50	+0.30	−0.1	440

**Table 2:** EDS Chemical composition analysis of AA2014 samples under different conditions before corrosion (wt.%).

Sample Designation				Before Corrosion							
	Al	Cu	Mn	Fe	Mg	Si	O	Na	Cl	Ca	K
BM	93.1	3.9	0.7	0.5	0.7	1.1	-	-	-	-	-
BP50	35.3	0.5	-	0.3	-	14.2	48.3	0.6	0.4	0.3	0.2
SiC/UP30	25.5	-	-	0.8	0.4	21.4	49.9	1.1	0.3	0.5	0.1
SiC/BP50_1000	21.3	-	-	0.2	0.4	23.7	52.3	1.5	0.1	0.3	0.2
SiC/BP50_2500	33.4	-	-	-	-	17.2	47.1	1.1	0.7	0.3	0.3

**Table 3:** Electrochemical parameters: corrosion potential (E_corr_), corrosion current density (Icorr), and anodic (βa) and cathodic (βc) slopes obtained from the Tafel plots through extrapolation.

Condition	βa (mV.dec^−1^)	−βc (mV.dec^−1^)	E_corr_ (V/SCE)	I_corr_ (µAcm^−2^)	Corrosion Rate Mpy
AA2014–BM	12.6	46.7	−0.664	2.276	1.279
AA2014–BP50	51.9	127.8	−0.768	0.624	0.0086
AA2014–SiC/UP30	7.6	108.8	−0.616	0.27	0.0037
AA2014–SiC/BP50_1000	36.5	96.1	−0.656	0.148	0.0020
AA2014–SiC/BP50_2500	64.4	32	−0.656	0.098	0.0013

**Table 4:** EIS fitting results of the AA2014 studied conditions in 0.5 M NaCl solution.

Condition	R_s_ (Ω.cm^2^)	CPC1 (Ω^−1^·s^n^·cm^−2^)	R_p_ (Ω·cm^2^)	CPC2 (Ω^−1^·s^n^·cm^−2^)	R_ct_ (Ω·cm^2^)
BM	8.377	27.92 × 10^−6^	-	-	7.902
BP50	26.19	8.883 × 10^−6^	-	-	32.387
SiC/UP30	42.85	4.415 × 10^−6^	168,904	3.944 × 10^−6^	214,938
SiC/BP50_1000	12.05	0.1518 × 10^−6^	4803	1.303 × 10^−6^	604,942
SiC/BP50_2500	39.97	42.87 × 10^−6^	14,130	25.36 × 10^−6^	659,404

## Data Availability

All the raw data supporting the conclusion of this paper were provided by the authors.
